# Evidence-based interventions for bipolar disorder: an open-access platform to guide treatment decisions based on efficacy, safety, and patient preferences.

**DOI:** 10.1192/j.eurpsy.2025.583

**Published:** 2025-08-26

**Authors:** M. De Prisco, V. Oliva, C. Gosling, S. Cortese, F. Jess, E. Vieta, M. Solmi

**Affiliations:** 1 Bipolar and Depressive Disorders Unit, FRCB-IDIBAPS , Barcelona, Spain; 2 DysCo Lab, Université Paris Nanterre, Nanterre; 3 Laboratoire de Psychopathologie et Processus de Santé, Université de Paris, Boulogne-Billancourt , France; 4 Centre for Innovation in Mental Health, School of Psychology, Faculty of Environmental and Life Sciences, University of Southampton, Southampton, United Kingdom; 5 Department of Psychiatry, Ottawa Hospital Research Institute, University of Ottawa, Ottawa, Canada

## Abstract

**Introduction:**

Bipolar disorder (BD) is a psychiatric condition characterized by a wide range of symptoms, for which many interventions have been proposed. Although the literature contains numerous meta-analyses (MAs) and network meta-analyses (NMAs) summarizing this evidence, the large volume and variability of information can make it difficult to apply in daily clinical practice, guide researchers, or inform the development of clinical guidelines.

**Objectives:**

To offer a freely accessible platform, developed within the U-REACH framework, providing updates on the latest therapeutic strategies for BD in terms of efficacy and safety, aligned with patient preferences. Informed by a living umbrella review, the platform aims to grade the current evidence to support informed decision-making.

**Methods:**

We conducted an umbrella review by searching PubMed, PsycINFO, and the Cochrane database up to December 31, 2023, for (N)MAs of randomized controlled trials investigating interventions for BD. Each association was assessed using the GRADE. Effect sizes were standardized into equivalent standardized mean differences (eSMD), with eSMD>0 indicating clinically positive effects and eSMD<0 indicating clinically negative effects. The results are made available on an open-access online platform. Users can filter data by age group, BD stage, intervention, effect size, outcome, comparison, type of meta-analysis, GRADE evidence level, and (N)MA quality. For any filter combination, users can visualize key interventions, outcomes, and a forest plot with eSMD. The database will be regularly updated. Additionally, a preference-based tool allows users to rank safety outcomes by importance (0-10) and the system will recommend medications based on these preferences.

**Results:**

From the 4,352 records retrieved, we included 71 (N)MAs evaluating the effects of pharmacological (n=87), brain stimulation (n=13), psychosocial (n=8), and circadian rhythm-based therapies (n=3), on 132 efficacy (n=85) and safety (n=47) outcomes. For the preference-based tool, we included 10 first-line interventions for at least one mood state of BD (aripiprazole, asenapine, cariprazine, lamotrigine, lithium, lurasidone, paliperidone, quetiapine, risperidone, valproate) and 15 safety outcomes based on clinical judgment (e.g., akathisia, weight increase, QTc prolongation, insomnia), resulting in 150 potential combinations.

**Conclusions:**

This platform represents a pioneering approach to delivering the most complete evidence on interventions for BD. With its regular updates, it provides clinicians and researchers with a freely accessible resource to guide treatment decisions based on efficacy, safety, and patient preferences. This tool aims to support the development of future guidelines, facilitate ongoing professional education, and ultimately improve the quality of care for individuals with BD.

**Disclosure of Interest:**

None Declared

